# Are *Ascaris lumbricoides *and *Ascaris suum *a single species?

**DOI:** 10.1186/1756-3305-5-42

**Published:** 2012-02-20

**Authors:** Daniela Leles, Scott L Gardner, Karl Reinhard, Alena Iñiguez, Adauto Araujo

**Affiliations:** 1 Departamento de Microbiologia e Parasitologia, Instituto Biomédico, Universidade Federal Fluminense, MIP-UFF, Rua Professor Hernani Melo 101, São Domingos, Niterói, 24210-130, RJ, Brazil; 2 School of Biological Sciences, University of Nebraska- Lincoln, Harold W. Manter Laboratory of Parasitology W 529 Nebraska Hall University of Nebraska-Lincoln Lincoln, Nebraska 68588-0514 USA; 3 School of Natural Resource Sciences, University of Nebraska at Lincoln, 6940 Van Dorn Street Ste 105, Lincoln, Nebraska 68506, USA; 4 Intituto Oswaldo Cruz, Fundação Oswaldo Cruz, Av. Brasil 4365, Manguinhos, Rio de Janeiro, 21045-900, RJ, Brazil; 5 Escola Nacional de Saúde Pública, Fundação Oswaldo Cruz, Rua Leopoldo Bulhões 1480, Manguinhos, Rio de Janeiro, 21041-210, RJ, Brazil

**Keywords:** *Ascaris*, coprolites, host-parasite evolution, paleoparasitology, parasitism, helminthiasis

## Abstract

Since the original description and naming of *Ascaris lumbricoides *from humans by Linnaeus in 1758 and later of *Ascaris suum *from pigs by Goeze 1782, these species have been considered to be valid. Four hypotheses relative to the conspecificity or lack thereof (and thus origin of these species) are possible: 1) *Ascaris lumbricoides *(usually infecting humans) and *Ascaris suum *(recorded mostly from pigs) are both valid species, with the two species originating via a speciation event from a common ancestor sometime before the domestication of pigs by humans, or 2) *Ascaris lumbricoides *in humans is derived directly from the species *A. suum *found in pigs with *A. suum *then existing as a persistent ancestor after formation of *A. lumbricoides*, or 3) *Ascaris suum *is derived directly from *A. lumbricoides *with the persistent ancestor being *A. lumbricoides *and *A. suum *being the newly derived species, and finally, 4) *Ascaris lumbricoides *and *A. suum *are the same species, this hypothesis being supported by studies showing both low morphological and low genetic divergence at several genes. We present and discuss paleoparasitological and genetic evidence that complement new data to evaluate the origin and evolution of *Ascaris *spp. in humans and pigs, and the uniqueness of the species in both hosts. Finally, we conclude that *Ascaris lumbricoides *and *A. suum *are a single species and that the name *A. lumbricoides *Linnaeus 1758 has taxonomic priority; therefore *A. suum *Goeze 1782 should be considered a synonym of *A. lumbricoides*.

## Review

Metazoan parasites of the order Ascaridida (Phylum Nemata: Class Secernentea) are classified into several families that occur in a wide range of hosts world-wide [1]. Mammals, from marsupials to human and non-human primates, birds, reptiles, and fishes, serve as common hosts [1-3]. Iguanodont coprolites dated from 100 million years ago were found positive for ascarid eggs [4]. Although found in many different hosts, species in this group are morphologically conservative, with little variation among groups [5].

*Ascaris lumbricoides *Linnaeus 1758 is a parasite of *Homo sapiens*, and *Ascaris suum *Goeze 1782 occurs in pigs (*Sus scrofa *Linnaeus 1758). These two ascarids were probably recognized by humans since prehistory, due to their abundance, adult size, symptoms, and distribution. Eggs are commonly found in coprolites, intestinal contents of mummies, and in other kind of archaeological material [6]. Because of their remarkable similarity, several hypotheses have been proposed to explain their origin in their respective hosts. 1) The first hypothesis would be: *Ascaris lumbricoides *(usually infecting humans) and *Ascaris suum *(recorded mostly from pigs) are both valid species. In this case, these two species would have originated via a speciation event from a common ancestor, probably sometime before the domestication of pigs by humans. There are no records of *Ascaris *from the great apes, so this hypothesis has little support. 2) *Ascaris lumbricoides *in humans is derived directly from the species *A. suum *found in pigs with *A. suum *then existing as a persistent ancestor. In this case the species *A. lumbricoides *could have arisen by an allopatric event of host-switching (pig to human). 3) *Ascaris suum *is derived directly from *A. lumbricoides *with the persistent ancestor being *A. lumbricoides *and *A. suum *being the more newly derived species. Exactly the opposite of 2 above. Finally, hypothesis 4) states that: *Ascaris lumbricoides *and *A. suum *are conspecific, this hypothesis has support from studies showing both low morphological and low genetic divergence (low genetic distances) in several genes. *Ascaris lumbricoides *is considered a parasite with a relatively long history of association with *Homo sapiens *[7,8] and up to the present time, neither classical taxonomony or molecular genetics have been able establish whether there is one or two distinct species. However, a particular host affiliation has been proposed [9-11]. Concepts and theories concerning the origin of *Ascaris *spp. parasitic in human and pig hosts will be briefly reviewed.

### Parasitological Evidence

Probably the main reason for two accepted species, *A. lumbricoides *and *A. suum*, was the finding of adult worms in the intestine of two distinct hosts, humans and pigs. The eggs found in fecal material are identical morphologically, but adult worms of the two species have slight differences in morphological characteristics that can be used to distinguish between them [12]. Attempts at experimental infections were successful in establishing the nematodes in pigs with worm eggs collected from humans and vice-versa and the parasite life cycle was completed in both hosts [13-17] ([see [11]]). An accidental laboratory human infection with *Ascaris *eggs extracted from pigs was also reported [18]. Nevertheless, in another separate experiment *Ascaris *eggs with the typical human-specific genotype were unable to mature in pigs [19]. It has also been shown that if cross infections from humans to pigs is attempted, it appears that a very heavy infective parasite load is needed to achieve infection in the pig hosts [7]. Therefore, there is still no consensus on infection potential in these two species of *Ascaris*.

The hypotheses of host switching and subsequent speciation presented above have also been proposed to explain the origin of *Trichuris trichiura *in humans and *Trichuris suis *in pigs [20]. However, *T. trichiura *in humans is now considered a so-called "heirloom species" that is shared by humans and apes and was inherited from a common primate ancestor [21]. Three aspects of the biology of species of *Trichuris *support this hypothesis [20]: a) *Trichuris *spp. are found in Old World non-human primates, and the most common species are *T. lemuris *in Lemuroidea, *T. cynocephalus *and *T. presbyticus *in Cercopithecoidea, and *T. trichiura *in Hominoidea. This last parasite was dispersed to other parts of the world via migrations of pre-historic humans; b) parasite ecological adaptations in the host - considering the shorter pre-patent period, egg maturation, and the longer lifespan of *T. trichiura *adult worms in relation to *T. suis, T. trichiura *is better adapted to the human host than is *T. suis *to pigs; c) paleoparasitological data showed *T. trichiura *eggs in archaeological material of human origin long before pig domestication [6].

When these aspects are examined regarding *Ascaris *spp. of humans and pigs, the facts generate some degree of controversy. *Ascaris *species are less diversified in Old World primates compared with *Trichuris *spp. and, relative to ecological adaptations adult worms of *A. lumbricoides *in humans have a longer lifespan, suggesting a possible better adaptation to humans than *A. suum *to pigs. However, egg maturation and prepatent periods of *A. suum *in pigs are shorter compared with *A. lumbricoides *in humans

Success in experimental infections or the presence of natural infections in other hosts, such as rodents, dogs, and non-human primates, among others, were recorded both for *A. suum *and/or *A. lumbricoides *([19,22-30] see [11]). There are also records of infections in non-human primates living in natural or similar conditions [23,26,29]. In most cases, infection was diagnosed by fecal parasitological analysis, and therefore it was not possible to distinguish between *A. suum *or *A. lumbricoides *infection, as it is impossible to distinguish the two species by their eggs (see above). Thus, the origin of *Ascaris *infection is uncertain where primates are in close contact with wild pigs. South American wild pigs (*Tayassu pecari *(Link, 1759) and *Pecari tajacu *(Linnaeus 1758)) were also found infected by *Ascaris *sp. [31,32]. However, these findings must be suspect, as they may reflect cross-infection from domestic pigs or even humans.

### Paleontological, Archaeological and Paleoparasitological Evidence

The oldest finding of *Ascaris *spp. eggs in humans was recorded from archaeological material dated 30,000 years before present (BP) [33], many thousands of years before pig domestication at 10,000 years ago [34]. In this study, there was no evidence of pigs in the archaeological context, but the organic sediment was also not clearly confirmed to be of human origin. Samples were collected from a cave inhabited in the past by both modern humans and cave bears, since *Ursus spelaeus *bone remains were identified. Although modern bears are infected by ascarids, the authors concluded that eggs were of human origin [33]. Therefore, the diagnosis was that of *A. lumbricoides *eggs based on the archaeological context. Further studies are needed to confirm this extreme antiquity for *A. lumbricoides*. These findings remain the oldest record for a putative *A. lumbricoides *human infection. Therefore, more samples should be submitted to paleoparasitological analysis to corroborate these data, and the ancient parasite-host association.

Humans and pigs appeared as species millions of years before the domestication of pigs. The infraorder Suina consists of species allocated to two families, Suidae and Tayassuidae. Paleontological evidence suggests that the Suidae diversified by the end of Oligocene (23 million years ago) in Europe, Asia, and Africa, with members of the family Tayassuidae appearing in North and South America by the end of Eocene (34 million years ago [35]. The emergence of great apes date between 24 to 16 myrs, and molecular data point to a divergence of human ancestors and apes estimated to 9 to 7 myrs [35,36]. Thus, human and non-human primates and wild pigs occupied the same environment long before domestication of pigs occurred, potentially favoring parasite transfer from one host to another. Wild pigs were hunted by humans since the begining of the Eocene, but closer contact was established with domestication [34].

**Figure 1:**
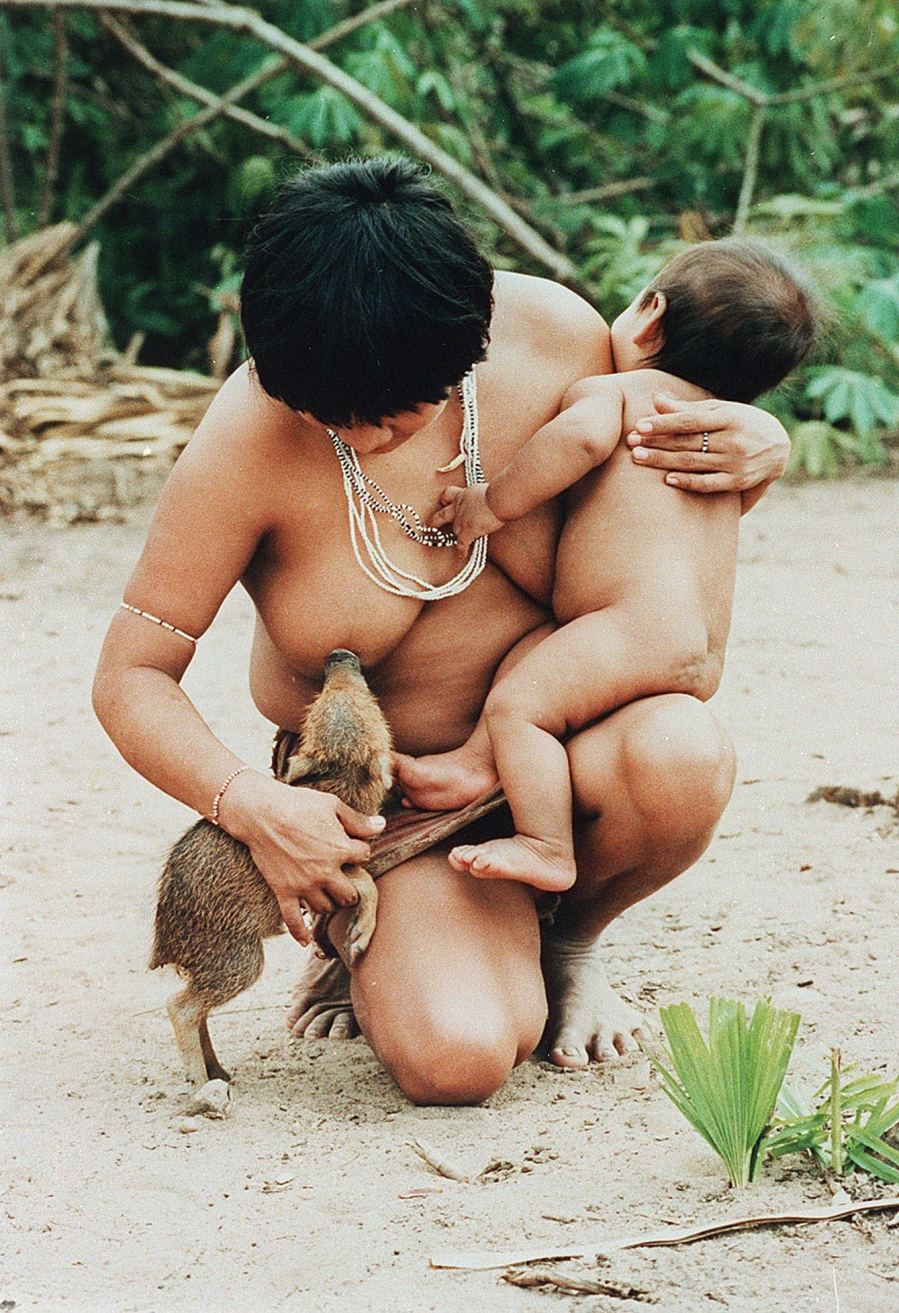
**Native Guajá from northeastern Brazil breastfeeding a wild pig**. Source: Pisco Del Gaiso, Folha de São Paulo - Brazil, 1992. The authors have received permission from the copyright holders Pisco Del Gaiso, Folha de São Paulo - Brazil to reproduce the image in this publication.

**Figure 2:**
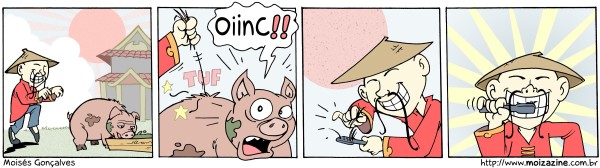
**Toothbrush origin**. The authors confirm that they have received permission from Moises Goncalves to reproduce the image in this publication. The image was originally published in http://www.moizazine.com.br.

After domestication of pigs occurred, the contact among hosts favored transference of *Ascaris *sp. of human and pig origin, promoting adaptations to one or other host. Contemporary examples of this close contact have been recorded in South American Indian communities [37], where suckling New World wild pigs (*T. pecari, P. tajacu*) are adopted and fed by women (Figure 1). Some Indian groups include rituals in which pig behavior is incorporated and acted out by young warriors [38]. There are ancient cultural and religious representations of close contact of humans and pigs in Europe and Asia [34,39], including an ancient toothbrush made of pig bristles (Figure 2).

Considering the above discussions, ascarids have been found today both in wild pigs and non-human primates in natural or close to natural conditions [23,26,29]. This could have happened also in the past.

### Evidence from Molecular Biology

The first attempts to separate the two species of *Ascaris *of human and pig origin aimed to identify specific molecular markers. Studies were mainly conducted in China, where high prevalence rates are found in both pigs and humans, and pig feces are used as fertilizers [9,10]. The interest on this subject increased after description of "cross infections" in North America and Denmark [9,18,40].

A zoonosis is characterized when the human host is infected by a parasite of wild or domestic animal [3]. Regarding *Ascaris *sp. in domestic pigs, due to the anthelmintic frequency and high doses used, cases of resistance have been registered in several animals [41]. Therefore, the zoonotic potential and possible cases of worm resistance may change public health strategies to control ascariasis in human communities [9,42].

Molecular epidemiology studies to distinguish the two species of *Ascaris *have been conducted [43]. PCR-RFLP (Polymerase Chain Reaction - Restriction Fragment Length Polymorphism) was applied to identify different restriction profiles for *Ascaris *sp. of both human and pig origin, using the nuclear region *ITS*1 (Internal Transcribed Spacer-1) as the molecular target [44]. In provinces in China, five parasite genotypes (G1-G5) for human and pig species were found using the same molecular target [45]. Although the G1 genotype is associated mainly with humans, and G3 occurs mostly in pigs, common *Ascaris *genotypes were found in both hosts. Intra-individual high variability in isolates of *Ascaris *sp. using as target the region *ITS*1 was found in Brazil, calling attention to the problems of using this target to genotype *Ascaris *spp. of human and pig origin [46].

Other molecular markers have been proposed and used to study molecular epidemiology of *Ascaris *spp. The mitochondrial markers (cytochrome c oxidase subunit 1 (*cox*1), and NADH dehydrogenase subunit 1 (*nad*1)) were used in samples from six Chinese provinces [47]. The authors found for the gene *cox*1, 10 different haplotypes of *Ascaris *spp. in the human host (H1-H10), and 10 for pigs (P1-P10). For *nad*1 11 different haplotypes of *Ascaris *spp. were found in the human host (H1-H11), and 15 in pigs (P1-P15). Although the authors considered a low genetic flow between these species, results showed a common haplotype for *Ascaris *sp. of human and pig origin for the *cox*1 gene. Microsatelites have also been used to study molecular epidemiology of *Ascaris *spp. showing that hybridization between worm population of human and pig origin probably occurred which may have implications for *Ascaris *control programs [42]. Recently, possible cases of human ascariasis from pig-derived *Ascaris *sp. were recorded in Japan [48]. Additionally, recent sequences available in Genbank from humans and non-human primates are identical to pig genotypes or haplotypes. In 2011 the complete mtDNA genome of *A. lumbricoides *was sequenced, showing that the complete *A. lumbricoides *mt genome differs from *A. suum *by only 1.9% [49]. Based on the high similarity of nucleotide and amino acid sequences of complete *A. suum *and *A. lumbricoides *mt genomes, the authors considered that both might represent the same species.

### Perspectives or utopia

The origin of taeniid tapeworms found in humans, including *Taenia solium, T. saginata*, and *T. asiatica *was tested by application of phylogenetic systematic methods of DNA sequences [50]. Human tapeworm species are similar to the species found in felids, wild canids, and African hyenas. The typical life cycle of the taeniids involves a carnivorous definitive host and an herbivorous intermediate host; tapeworm eggs eliminated by these carnivores in the feces are ingested by herbivores. Hominids became infected, and served well as the definitive host, by eating herbivores infected with tapeworm larva. The adults usually occurred in other sympatrically occurring scavenging carnivores such as hyenas. After switching hosts, a speciation event occurred and humans maintained their own two species of *Taenia*. Therefore, these cestodes of humans adapted to pigs and cattle as intermediate hosts sometime after domestication of these animals. Molecular biology studies suggest that *Homo erectus *introduced *T. asiatica *in Asia, and the other human species of *Taenia *were introduced to the Americas only after Columbus [51-53]. Genetics and paleoparasitological data support this hypothesis due to the fact that no *Taenia *sp. eggs have yet been found in pre-Columbian South American archaeological material.

Considering the data presented above, "the comparative method" would call for a broad study of the genetics of *Ascaris *spp. in modern humans and pigs from different regions, we also recommend that a broad survey of other vertebrate hosts be conducted following the *Taenia *sp. model (see methods in Gardner [54] and Gardner and Jimenez-Ruiz [55]). In *Ascaris *sp. studies it is possible to include New World and Old World archaeological material [6], and recover ancient parasite DNA to compare with modern sequences.

Molecular paleoparasitology techniques have been applied to *Ascaris *spp. [56-58]. However, to accumulate solid evidence more samples should be examined, as well as other molecular targets. Another difficulty is that human coprolites are found in archaeological sites in greater number than pig coprolites.

Phylogenetic relationships of nematodes classified in the Ascaridoidea were estimated by Nadler and Hudspeth [59] based on a total evidence parsimony analysis of a combination of morphology and one mitochondrial and two nuclear genes. Their study shows that *A. lumbricoides *and *A. suum *are sister taxa that share a most recent common ancestor with *Parascaris equorum *the large ascarid of horses. Because these species share a most recent common ancestor, there is no way to place either *A. suum *or *A. lumbricoides *in a position as "the more derived species". Nadler and Hudspeth [59] note that in their study, *A. lumbricoides *had 7 unambigious molecular autapomorphies while *A. suum *had one rDNA autapomorphy and that the level of genetic differentiation (genetic distance) was low, showing the smallest pairwise distance among all taxa included in their analysis.

## Conclusions

The origin of *Ascaris *spp. is still not well understood. Results obtained from experimental infections and molecular methods have been inconclusive, and before this time, none of the published studies answered the question of whether *A. lumbricoides *and *A. suum *are truly distinct species.

Mitochondrial markers in modern parasite samples from humans and pigs from Brazil, collected in rural and urban areas, as well as in American Native Indian communities, were analyzed. All samples were collected from people in close contact with pigs and data showed common haplotypes in *Ascaris *sp. derived both from human and pig hosts. Taking into account the history of close contact of humans and wild pigs, estimated at about 10,000 years before present, the verified nematode crossinfections between humans and pigs, and the documented hybridization that occurred between *A. lumbricoides *and *A. suum*, together with the recent insights about high levels of genetic similarity between the complete mtDNA genomes of *A. lumbricoides *and *A. suum *[6,9-11,18,40,42,59] we conclude that only a single interbreeding population of *Ascaris *exists, and the genotypic and phenotypic differences are only manifested at the population level. The populations occurring in humans or pigs have only slight phenotypical and genotypic adaptative changes; however, with a single natural history. Thus, we recommend synonymizing these two species, with the name *Ascaris lumbricoides *Linnaeus 1758 taking priority over *Ascaris suum *Goeze 1782.

## Competing interests

The authors declare that they have no competing interests.

## Authors' contributions

DL wrote the first draft, and DL and AMI were responsible for molecular biology studies; AA, SLG, and KR discussed paleoparasitology, parasitism, and roundworm and whipworm evolution; DL was responsible for the main results; all authors approved the final version of the manuscript.

## Authors' information

DL, Sc.D., is Associate Professor, publishing on molecular biology and paleoparasitology; AMI, Sc.D., is a Research Assistant, publishing on molecular biology and paleoparasitology; SLG, Ph.D., Curator and Professor, Harold W. Manter Laboratory of Parasitology, KR, Ph.D., Fulbright Commission Senior Specialist in Botanical Archaeology, and Professor in Forensic Science; AA, MD, Sc.D., Senior Researcher, paleoparasitology specialist since 1980.
